# The evaluation of three diagnostic tests for the detection of equine influenza nucleoprotein in nasal swabs

**DOI:** 10.1111/irv.12235

**Published:** 2014-02-07

**Authors:** Pamela Galvin, Sarah Gildea, Maura Nelly, Michelle Quinlivan, Sean Arkins, Cathal Walsh, Ann Cullinane

**Affiliations:** a Virology Unit, The Irish Equine CentreJohnstown, Naas, Co. Kildare, Ireland; b Department of Life Sciences, University of LimerickLimerick, Ireland; c Department of Statistics, Trinity CollegeDublin, Ireland

**Keywords:** Diagnosis, ELISA, equine influenza, Espline, nucleoprotein Directigen, rapid antigen detection

## Abstract

**Background:**

Equine influenza (EI) is a highly contagious respiratory disease of horses.

**Objectives:**

The aim of this study was to evaluate two rapid antigen detection kits (Directigen or DFA, and Espline) and a commercial ELISA for the detection of EI nucleoprotein in nasal swabs.

**Method:**

Nasal swab samples from naturally and experimentally infected horses were used to compare the sensitivity and specificity of these assays to virus isolation (VI) and real-time RT-PCR.

**Results:**

If real-time RT-PCR was considered as the gold standard, the sensitivity of the other tests in field samples was 68% (DFA), 35% (ELISA), 29% (Espline), and 9% (VI). These tests had 100% specificity when compared to real-time RT-PCR. A receiver operating characteristic (ROC) curve indicated that decreasing the cutoff of the ELISA would increase sensitivity with some loss of specificity. In samples from experimentally infected horses, the sensitivity of the tests compared with real-time RT-PCR was 69% (VI), 27% (DFA), 6% (Espline), and 2% (ELISA). The specificity was 100% for Espline and ELISA and 95% for VI and DFA.

**Conclusions:**

This study illustrated that DFA is the most sensitive antigen detection test evaluated for the diagnosis of EI and that it can detect virus in some subclinical infected and vaccinated horses. The results suggest that DFA is a useful adjunct to laboratory tests and may be effective as a screening test in a quarantine station or similar facility where horses are monitored daily.

## Introduction

Equine influenza (EI) is a highly contagious respiratory disease of horses caused by an RNA virus of the Orthomyxoviridae family.^1^ Influenza viruses are classified on the basis of the composition of the surface glycoproteins hemagglutinin (HA) and neuraminidase (NA). Although avian influenza H5N1 has been associated with respiratory disease in donkeys in Egypt,^2^ all other outbreaks of EI that have been reported for over three decades have been due to H3N8 viruses. To date, EI outbreaks have occurred all over the world with the exception of a small number of island nations including New Zealand and Iceland. The importation of subclinically infected vaccinated horses and inadequate quarantine procedures have resulted in several major outbreaks of EI in susceptible populations, for example South Africa (1986 and 2003),^3^,^4^ India (1987),^5^ Hong Kong (1992)^6^, and Australia (2007).^7^

The introduction of a single infected horse can result in an explosive virus spread in unprotected horses over a wide geographical area. Rapid diagnosis, movement restrictions, and vaccination are the key control measures for EI. A definitive diagnosis of EI can only be made by isolation or detection of the virus from/in nasopharyngeal swabs or by serological examination of paired serum samples. EI may be isolated in embryonated hens' eggs or less frequently, in Madin–Darby canine kidney cells.^8^ Virus isolation (VI) is necessary for virus characterization and strain surveillance, but as a diagnostic technique, it has largely been supplanted by ELISA, RT-PCR, or real-time RT-PCR. Real-time RT-PCR is the test of choice in most laboratories as it is highly sensitive and provides a diagnosis in hours.^9^–^12^ However, commercial rapid antigen detection (RAD) kits for the diagnosis of human influenza have been used in the diagnosis of EI.^13^–^18^ These kits are all based on the binding of influenza A viral nucleoprotein (NP) to antibody that is specific for this highly conserved protein. They have been used for diagnosis during outbreaks and to screen imported horses in quarantine. The main objective of this study was to compare the sensitivity of two of these kits and that of a commercial ELISA for the detection of influenza A viral nucleoprotein in pigs, birds, and horses, to VI and real-time RT-PCR.

## Materials and methods

### Nasopharyngeal swabs

Nasopharyngeal swabs were collected from 64 horses on premises where EI was diagnosed by real-time RT-PCR and VI and/or serology. The premises included a polo yard (*n* = 16), three racing yards (*n* = 27), a non-Thoroughbred yard (*n* = 6), a showjumping yard (*n* = 5), a Thoroughbred stud (*n* = 6), and a non-Thoroughbred stud (*n* = 4). Following sample collection, nasopharyngeal swabs were placed in 5 ml of viral transport medium as previously described.^19^ Samples were tested by real-time RT-PCR and stored at −70°C until tested by additional methods.

Nasopharyngeal swabs were also collected from seven seronegative horses on the day before and daily for 14 days post-exposure to an aerosol of 10 ml A/eq/Kildare/89 at 10^6^ 50% egg infective dose (EID_50_)/ml as described previously.^20^ Samples were tested by real-time RT-PCR and VI and stored at −70°C until tested by additional methods.

### Directigen Flu A

Directigen Flu A (DFA), an *in vitro* enzyme immunoassay membrane test (Becton, Dickinson and Company, Sparks, MA, USA), was used in accordance with the manufacturers' instructions and as previously described.^9^ In this study, the degree of positive reaction was scored from 1 to 3 with 1 being a dark purple triangle on the test device (strong positive), 2 a light-colored triangle on the test device (medium positive), and 3 an outline of a triangle on the test device (weak positive).

### Espline influenza A&B-N

Espline Influenza A&B-N (Espline) an immuno-chromatography cassette-style test using anti-influenza type A and B virus monoclonal antibodies (Fujirebio Inc, Tokyo, Japan) was used in accordance with the manufacturers' instructions. Each nasopharyngeal swab was soaked in the extraction solution provided. Two drops of the sample diluted in the extraction fluid (approximately 30 μl) were dropped onto the sample window which contains alkaline phosphatase-labeled monoclonal antibody against influenza virus nucleoprotein. Antigen antibody complexes migrated to fixed antibody where a positive sample was indicated by the production of a blue line on addition of substrate. In this study, the intensity of the line was graded from 1 to 3, with 1 being a strong positive.

### ID screen influenza A antigen capture ELISA

ID Screen Influenza A Antigen Capture ELISA (ELISA), which is used to detect influenza A viral nucleoprotein, was carried out in accordance with the manufacturer's instructions (IDvet, Montpellier, France). Briefly, the wells of the test plate were coated with anti-Antigen A monoclonal antibody. Nasopharyngeal samples were diluted 1:2, added to the test wells, and incubated at 37°C for 60 minutes. Bound antigen was detected with peroxidase-labeled antibody.

### VI and quantification

Nasopharyngeal swabs were passaged up to six times in the allantoic cavities of 9- 12-day-old embryonated hen's eggs as described previously.^19^ Allantoic fluid was tested for hemagglutinating activity using 1% hen red blood cells.^8^ If hemagglutination was observed, the virus isolate was typed by hemagglutination inhibition (HI) using type-specific ferret antisera supplied by the National Institute of Biological Standards, England. Quantification assays to determine the EID_50_ of nasopharyngeal swabs collected following experimental infection were carried out and results calculated in accordance with standard procedure.^21^

### Real-time RT-PCR

RNA was extracted from 100 μl nasopharyngeal samples collected from experimentally infected horses using the RNAgents Total RNA Isolation System (Promega Corporation, Madison, WI, USA) in accordance with the manufacturer's instructions. One-step real-time RT-PCR was performed using the Light Cycler RNA Amplification kit, SYBR Green I (Roche, Burgess Hill, West Sussex, UK) as previously described.^10^

RNA was extracted from 140 μl nasopharyngeal swabs submitted from clinical samples using the QIAamp Viral RNA Mini Kit (Qiagen, Crawley, West Sussex, UK) in accordance with the manufacturer's instructions. One-step real-time RT-PCR using a primer probe-based assay which targets the matrix gene of influenza A virus^22^ and an AgPath-ID One-Step RT-PCR Kit (Ambion, Austin, TX, USA) on an ABI 7500 Fast thermal cycler (Applied Biosystems, Austin, TX, USA) platform was carried out. Briefly, 5 μl of purified nucleic acid was added to a 20 μl reaction mix containing 25× RT buffer, 80 ng tRNA (Laborchemikalien GmbH, Seelze, Germany), 0·36 μm of each primer, 0·15 μm of probe and 25× RT enzyme. One-step RT-PCR was carried out at 45°C for 10 minutes followed by 95°C for 10 minutes, 45 cycles of 95°C for 15 seconds and 60°C for 60 seconds.

### Hemagglutination inhibition

Sera were tested for antibodies against A/eq/Prague/56 (H7N7), A/eq/Kildare/89 (H3N8 – European lineage), and A/eq/Kildare/92 (H3N8 – American lineage) using the HI test in accordance with standard procedure and as previously described.^8^,^19^ Seroconversion was defined as a fourfold or greater increase in antibody titer.

### Statistical analysis

The evaluation of the diagnostic tests was undertaken separately in experimental and clinical samples. In order to examine symmetry in classification as positive or negative between the different assays, a two-way classification table was constructed. Symmetry was then measured using the McNemar test, and associated chi-square statistic and *P*-values were obtained. The association between the DFA and EID_50_ was examined using the Mann–Whitney *U*-test. To examine the optimum ELISA cutoff value using real-time RT-PCR as the gold standard, a receiver operating characteristic (ROC) curve was plotted and area under the curve (AUC) statistic calculated. Analysis was carried out using R Studio running r version 3.0.1, (http://www.r-project.org).

## Results

**Figure 1:**
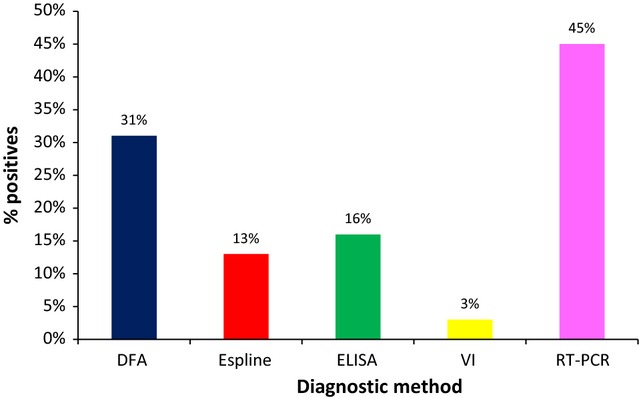
Comparison of EI detection methods in nasopharyngeal swabs (*n* = 75) from naturally infected horses. EI, Equine influenza.

Seventy-five nasopharyngeal samples collected from 64 horses were tested for EI by DFA, Espline, ELISA, VI, and real-time RT-PCR. The horses were located on eight different premises where EI was confirmed by laboratory testing. The percentage of positives detected by each test is summarized in Figure 1.

Virus was isolated from two samples on repeat passage in embryonated eggs. Real-time RT-PCR detected the highest number of positive samples. The nasal swab samples from horses 1 and 54 (Table 1) were only positive by real-time RT-PCR, but the results were confirmed using the Light Cycler real-time RT-PCR assay. Real-time RT-PCR-negative samples tested negative by all other assays. If real-time RT-PCR was considered as the gold standard, the sensitivity of the other tests was 68% (DFA), 35% (ELISA), 29% (Espline) and 9% (VI). These tests had 100% specificity when compared to real-time RT-PCR. Examination of positive versus negative results indicated that there was significant disagreement between PCR, and all other assays included in this study (*P* < 0·01). Of the three tests under evaluation, DFA was significantly more sensitive than Espline (*P* < 0·001) and ELISA (*P* < 0·001), but there was significant agreement between the latter two assays.

**Table 1:** Detection of EI in nasopharyngeal swabs from naturally infected horses

Horse	Clinical signs	Vaccination	DFA	Espline	ELISA	VI	Real-time RT-PCR	SC
1	+	−	−	−	−	−	+	−
5	+	−	+(3)	+(1)	−	−	+	+
6	+	−	+(1)	−	−	−	+	+
8	+	−	+(3)	+(1)	−	−	+	+
10	−	−	+(1)	−	−	−	+	−
11	−	−	+(3)	+(1)	+	−	+	N/A
12	−	−	+(1)	−	−	−	+	+
17	+	−	−	−	−	−	+	−
21	+	+	+(3)	−	+	−	+	−
22	+	−	+(1)	−	−	−	+	+
23	+	+	+(3)	+(2)	+	−	+	+
24	+	+	+(2)	−	+	−	+	+
25	+	+	+(1)	−	−	−	+	−
26	+	+	+(2)	−	+	−	+	+
27	+	−	+(1)	−	−	−	+	+
28	+	−	+(1)	−	−	−	+	+
34	−	+	+(1)	−	−	−	+	+
40	+	Unknown	−	−	−	−	+	N/A
41	−	Unknown	−	−	−	−	+	N/A
43	−	Unknown	−	−	−	−	+	N/A
45	+	Unknown	+(3)	+(2)	+	−	+	+
46	−	Unknown	−	−	−	−	+	N/A
48	+	Unknown	+(3)	+(1)	+	−	+	+
49	+	+	+(3)	+(1)	+	+	+	+
50	+	+	+(2)	−	−	+	+	+
54	+	−	−	−	−	−	+	−
57	−	+	+(2)	−	+	−	+	−
61	+	Unknown	+(3)	+(2)	+	−	+	+
63	+	Unknown	+(3)	+(2)	+	−	+	N/A
64	+	Unknown	+(3)	+(2)	+	−	+	N/A
67	−	+	−	−	−	−	+	−
68	−	Unknown	−	−	−	−	+	−
69	−	+	−	−	−	−	+	−
70	−	+	−	−	−	−	+	−

DFA, Directigen Flu A; EI, equine influenza; SC, seroconversion to H3N8; N/A, not applicable as no convalescent sample received; VI, virus isolation.

Clinical signs = presence of one or more of the three most common clinical signs associated with influenza, that is, pyrexia, nasal discharge, coughing.

The positive samples detected by ELISA and Espline were all detected by DFA. Furthermore, all Espline positives and nine of 12 ELISA positives were detected as strong positives (3) by DFA (Table 1). Thirty-four of the 75 samples were detected as positive by one or more method. Seven swabs were positive by real-time RT-PCR and the three commercial kits, but no virus was subsequently isolated. Only one horse, horse 49 was detected as a positive by all five methods (Table 1). Twelve positive samples detected by real-time RT-PCR were from subclinical infected horses. Five of these were detected by DFA, two by ELISA, and only one by Espline. Of the nine positive samples from vaccinated horses detected by real-time RT-PCR, seven were detected by DFA, five by ELISA, and only one by Espline.

### Selection of optimum ELISA cutoff by ROC analysis

**Figure 2:**
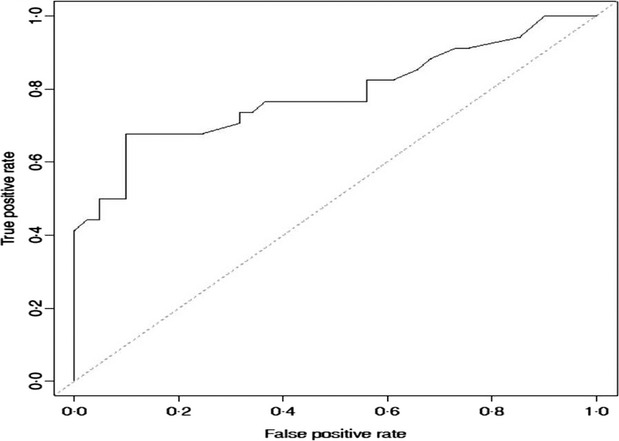
The selection of ELISA cutoff points by ROC analysis. ROC, receiver operating characteristic.

For these data, alternative values of the multiplier to classify positive or negative results were examined. The performance of this test is summarized by the ROC curve (Figure 2).

Decreasing the ELISA cutoff from four times the mean O.D. of the negative control to two times the mean O.D. of the negative control increased the sensitivity from 35% to 50% of real-time RT-PCR in clinical samples while only decreasing the specificity by 5%. Decreasing the ELISA cutoff from four times the mean O.D. of the negative control to 1·25 times the mean O.D. of the negative control increased the sensitivity to 65% of real-time RT-PCR in clinical samples, but decreased the specificity by 12%.

### Detection of EI in post-experimental infection samples

**Figure 3:**
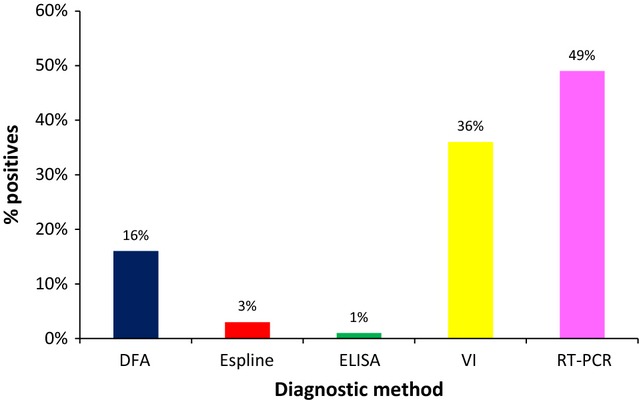
Comparison of EI detection methods in nasopharyngeal swabs (*n* = 104) from experimentally infected foals. EI, Equine influenza.

**Figure 4:**
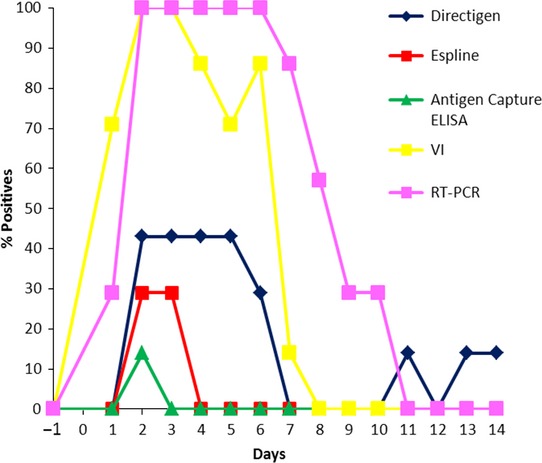
Detection of EI in nasopharyngeal swabs (*n* = 104) collected from foals on day −1 to day 14 post-infection. EI, Equine influenza.

The results of the detection of EI in nasopharyngeal swabs from experimentally infected foals by VI, real-time RT-PCR, and the three commercial antigen detection kits are summarized in Figures3 and 4. All foals seroconverted post-challenge. If real-time RT-PCR was considered as the gold standard, the sensitivity of the other tests was 69% (VI), 27% (DFA), 6% (Espline), and 2% (ELISA). The specificity of these tests compared with real-time RT-PCR was 100% for Espline and ELISA and 95% for VI and DFA. Examination of positive versus negative results post-experimental infection indicated that there was significant disagreement between PCR and all other assays included in this study (*P* < 0·01). Of the three tests under evaluation, DFA was significantly more sensitive than Espline (*P* < 0·001) and ELISA (*P* < 0·001), but there was significant agreement between the latter two assays.

None of the antigen detection kits used were as sensitive as either VI or real-time RT-PCR post-experimental infection. Peak viral shedding occurred from day 2 to day 6 post-experimental infection (Table 2). Positives were detected by real-time RT-PCR from day 1 to day 10 with the majority of samples being identified as positive at the time of peak shedding. A gradual decrease in the number of positive samples detected by real-time RT-PCR was observed after day 6. Overall, real-time RT-PCR was the most sensitive method of detection.

**Table 2:** Mean EID_50_ of EI in nasopharyngeal swabs collected from day 1 to day 7 post-experimental infection

Day	1	2	3	4	5	6	7
No. of VI positives	5/7	7/7	7/7	6/7	5/7	6/7	1/7
Mean EID_50_/ml	10^0·9^	10^2·4^	10^2·3^	10^2·3^	10^3·0^	10^1·9^	10^1·5^
Standard error	10^0·19^	10^0·35^	10^0·22^	10^0·34^	10^0·47^	10^0·30^	N/A

EI, equine influenza; VI, virus isolation.

Directigen Flu A was the most sensitive of the two RAD kits (Figure 3). The EID_50_ of the DFA-positive samples ranged from 10^1·5^ to 10^4·5^ (Table 3). There was a significant association between the EID_50_ and the DFA results (*P* < 0·0001). Positives samples were detected by DFA on days 2 to 6 when virus shedding peaked. This kit also detected weak positives on days 11, 13, and 14, but no virus was isolated. Only three positive samples were detected by the Espline kit. The EID_50_ of the Espline-positive samples ranged from 10^3·5^ to 10^4^ (Table 3). Only one positive sample collected on day 2 post-experimental infection was detected with the ELISA. This positive was a grade 3 positive by DFA and a grade 2 by Espline and had a titer of 10^3·25^ EID_50_. Thus, the only positive sample detected with the ELISA was from an animal shedding a high concentration of virus.

**Table 3:** EID_50_ of EI in nasopharyngeal swabs positive by antigen detection

Horse	Day	DFA	Espline	ELISA	EID_50_/ml
B	2	Pos (1)	Neg	Neg	10^2·5^
F		Pos (3)	Pos (2)	Pos	10^3·25^
G		Pos (2)	Pos (1)	Neg	10^4^
D	3	Pos (1)	Neg	Neg	10^1·75^
E		Pos (1)	Pos (1)	Neg	10^3·5^
F		Pos (1)	Neg	Neg	10^2·25^
B	4	Pos (1)	Neg	Neg	10^1·5^
E		Pos (2)	Neg	Neg	10^3·5^
F		Pos (1)	Neg	Neg	10^2·5^
A	5	Pos (2)	Neg	Neg	10^4·5^
E		Pos (1)	Neg	Neg	10^1·75^
C	6	Pos (1)	Neg	Neg	10^1·75^
D		Pos (1)	Neg	Neg	10^1·75^

DFA, Directigen Flu A; EI, equine influenza.

### Sensitivity and specificity of VI, DFA, Espline, and ELISA when compared to real-time RT-PCR

Analysis of the combined results obtained with the samples from naturally infected horses and those from experimentally infected horses indicated that there was significant disagreement between real-time RT-PCR and all other assays included in this study (*P* < 0·001). If real-time RT-PCR was considered as the gold standard, the sensitivity of the other tests was 44% (VI), 44% (DFA), 15% (Espline), and 15% (ELISA). VI and DFA demonstrated a specificity of 97%, and Espline and ELISA were 100% specific compared with real-time RT-PCR.

## Discussion

The rapid and accurate detection of EI is essential if the laboratory diagnosis is to have a significant impact on the management of disease. Sensitive, specific, and rapid tests are necessary to ensure the isolation of infected horses and the prevention of transmission to susceptible horses, to prevent unnecessary treatment with antibiotics and to encourage vaccination in the wider population. These tests are also essential to monitor the status of vaccinated horses in quarantine to prevent the introduction of virus to susceptible populations. This study compared the sensitivity of two RAD kits and a commercially available ELISA to VI and real-time RT-PCR.

The results of the comparative studies in both naturally infected and experimentally infected horses confirmed that real-time RT-PCR is the most sensitive technique available for the detection of EI. This was consistent with previous studies.^9^,^10^,^16^,^23^ Testing of the clinical samples indicated that real-time RT-PCR is useful for the screening of subclinically infected vaccinated horses. However, positive results with the more sensitive real-time RT-PCR assays such as the probe-based assay used in this study to test the clinical samples do not always correlate with the presence of replicating virus.^23^ The examination of samples from outbreaks of EI in this study identified 11 horses that were positive by real-time RT-PCR that tested negative by all other virus detection tests. During the 2007 outbreak in Australia, it was demonstrated that horses may test positive by real-time RT-PCR long after there is a likelihood they are infectious and constitute a risk to other horses.^23^ RNA was detected up to 34 days after infection although experimental infection studies estimate that horses remain infectious for <14 days. Nonetheless, real-time RT-PCR is the test of choice to minimize the risk of EI incursions associated with horse movement, and the cycle threshold or *C*_t_ value of serial nasal swab samples along with clinical and epidemiological data may be used to interpret the significance of positive tests.

The RADs and the ELISA are similar to real-time RT-PCR in that they also detect a viral component rather than viable virus. The ELISA is marketed for the testing of birds, swine, and horses. DFA and Espline are marketed primarily for the detection of human influenza viruses, but both effectively detect non-human influenza A viruses^24^,^25^ and in a comparative study have been shown to be the most sensitive RADs for the detection of EI.^17^ In this study, DFA proved to be the most sensitive of the three tests in the examination of clinical and experimental samples from horses exposed to EI. Espline was slightly more sensitive than the ELISA for the detection of EI post-experimental infection, but this was reversed when testing clinical samples. The superiority of DFA was confirmed by comparing the limit of sensitivity of the three assays using known concentrations of virus (results not shown). The DFA was able to detect less than 1HA of virus, a result consistent with that reported by Chambers *et al*.^13^ This level of sensitivity was also evident with BD Directigen EZ Flu A + B which has replaced DFA since the study was completed. Espline and the ELISA had a limit of detection of 5·5 HA units of virus. The findings differ from those of Yamanaka *et al*.,^17^ who found similar detection limits for Espline and DFA in virus stock and almost equal sensitivities in the detection of virus in nasal swabs from three experimentally infected horses. However, the horses were older (2 years old as opposed to foals), and the challenge dose was greater (10^8·6^ EID_50_/ml compared with 10^6^ EID_50_/ml) which may have impacted on the results. Yamanaka *et al*.,^17^ also reported two apparent false-positive results with DFA and suggested that the specificity of Espline was superior to DFA. The specificity of Espline and of the ELISA was not called into question in this study as all samples detected as positive by one or both of these tests also tested positive by other assays. However, in the experimental study presented here, three weak positive samples were detected by DFA on days 11, 13, and 14 post-experimental infection. The time post-experimental infection and the fact that these positives were not detected by any other diagnostic method suggest that the veracity of the results is open to question. Thus, as with real-time RT-PCR results, it may be best to interpret weak DFA-positive results in conjunction with other data.

In the study presented here, the DFA test exhibited very different sensitivity compared with VI in eggs, in the analysis of field samples compared with the results obtained using samples from experimentally infected foals. The percentage of clinical samples detected as positive by DFA was over 30% compared with <3% by VI. In contrast, the percentage of samples from experimentally infected horses detected as positive by DFA was 16% compared with 36% by VI. This was consistent with previous studies by Chambers *et al*.,^13^ Quinlivan *et al*.,^9^ and Yamanaka *et al*.,^17^ who reported that DFA was less sensitive than VI for the detection of virus in experimentally infected horses. However, some virus strains are more readily isolated and propagated in eggs than others. The challenge virus used in this study A/eq/Kildare/89, a virus of the European lineage, was far easier to isolate in eggs than the viruses of the American lineage that have been responsible for the majority of the outbreaks in recent years.

It has been suggested that DFA is most useful at the peak of infection but less sensitive early or late in infection when low levels of virus are shed.^26^ In the experimental infection study, peak viral shedding occurred from day 2 to day 6 post-infection, and there was a significant association between the titer of virus in the nasal swabs and the DFA results (*P* < 0·0001). This suggests that the DFA will be most effective for the diagnosis of EI if the nasal swabs are collected from acutely infected horses. This is not always the case in the field where delayed veterinary intervention is commonplace.^19^,^27^ However, it is standard practice to monitor imported horses on a daily basis which may in part contribute to the success of RAD tests in quarantine facilities.

Directigen Flu A and Espline take approximately 15 minutes, require no specialized equipment, and can be performed by personnel that are not specially trained in virological techniques. In this study, DFA was found to be more sensitive than Espline. DFA was also shown to be more sensitive than the laboratory-based ELISA and simpler and more rapid to perform. The ELISA takes approximately 2 hours and is suitable for high-throughput testing. However, this study found a low rate of positive detection with this test, suggesting that it is insufficiently sensitive to accept negative results. Decreasing the ELISA cutoff from four times the mean O.D. of the negative control to two times the mean O.D. of the negative control increased the sensitivity to 50% of real-time RT-PCR in clinical samples while only decreasing the specificity by 5%, suggesting that altering the cutoff might improve the ELISA as a screening test in circumstances where there is limited access to other assays.

This study illustrated that DFA can detect virus in some subclinical infected and vaccinated horses, confirming that if real-time RT-PCR is not readily available, it could be used as a preliminary screen for horses in quarantine. DFA is used routinely to screen imported horses in quarantine in Dubai.^15^ In 2012, infected endurance horses imported from Uruguay into The Dubai Racing Club Quarantine tested positive by DFA, and the virus was subsequently isolated and characterized. RADs are also used to screen imported horses to Hong Kong, a practice introduced after the outbreak of EI in 1992.^6^,^28^ Recently some quarantine facilities have replaced DFA with Espline. Although Espline is easier to use, this study suggests that DFA is more sensitive and thus has superior potential for preventing an incursion of EI into a susceptible population. However, DFA and Espline are not a substitute for real-time RT-PCR. They are significantly less sensitive than real-time RT-PCR which is the most appropriate test for the international movement of horses. A positive result with an antigen detection system is a good indication of EI infection, but all suspect cases that test negative should be retested by real-time RT-PCR.
